# Obituary: Klaus Starke (1937–2024)

**DOI:** 10.1007/s00210-024-03040-8

**Published:** 2024-03-18

**Authors:** Lutz Hein, Klaus Aktories, Roland Seifert

**Affiliations:** 1 Institute of Experimental and Clinical Pharmacology and Toxicology, Faculty of Medicine, University of Freiburg, Albertstr. 25, 79104 Freiburg, Germany; 2 Institute of Pharmacology, Hannover Medical School, Hannover, Germany

**Figure:**
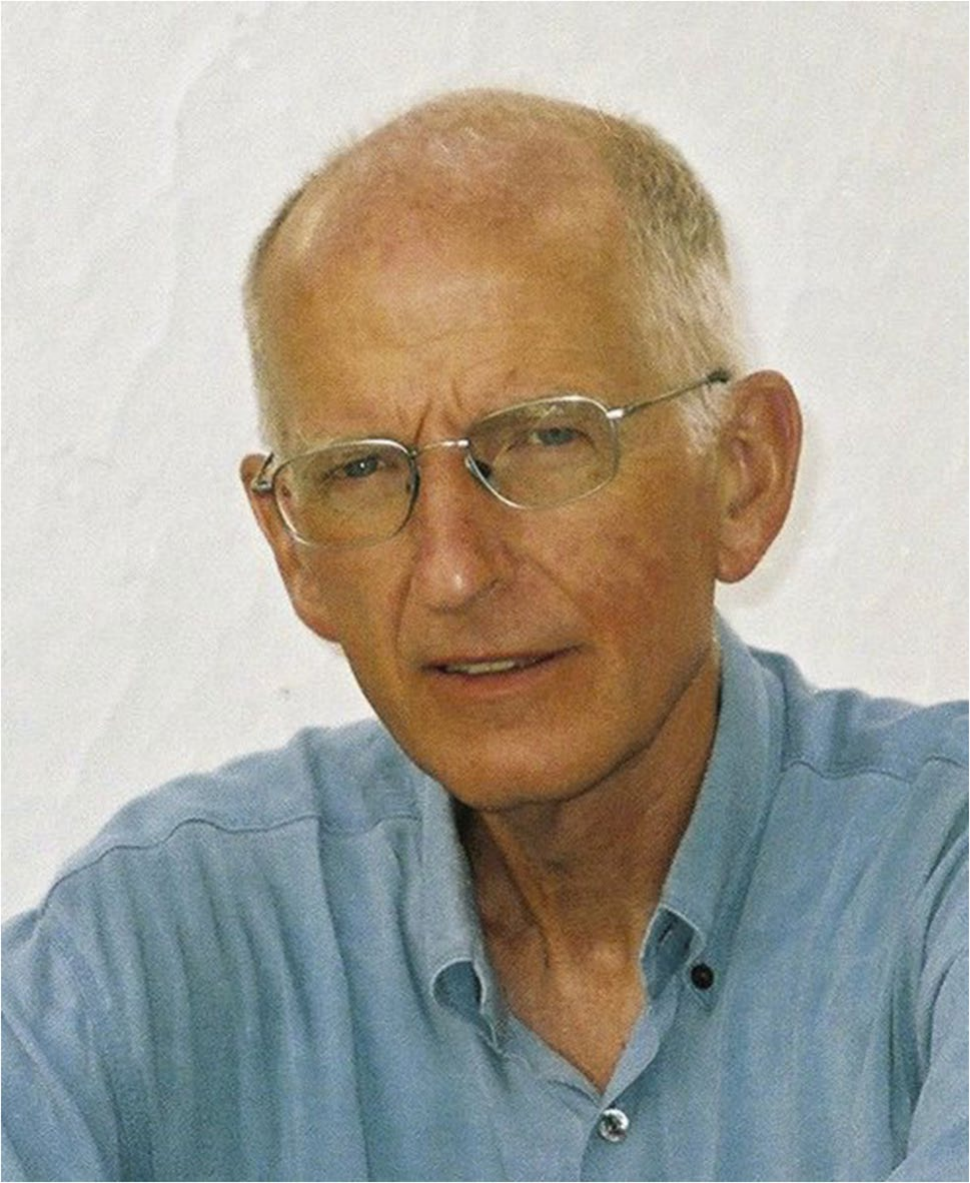
Klaus Starke (von Family Klaus Starke - Eigenes Werk, CC BY-SA 3.0, https://commons.wikimedia.org/w/index.php?curid=14930748)

Prof. Klaus Starke died in January 2024 at the age of 86 in Freiburg, Germany.

We lost an outstanding scientist and academic teacher whose work has left a lasting impact on the field of Pharmacology.

Klaus Starke studied pharmacy and medicine in Freiburg, Erlangen, Tübingen, and Heidelberg, Germany. In 1965, he received his doctoral degree in Tübingen with a thesis on substance P in the brain under the supervision of Prof. Fred Lembeck. The habilitation was awarded in Essen, Germany, where Klaus Starke opened a field of research that he subsequently strongly influenced—presynaptic receptors. He investigated how angiotensin acts on sympathetic nerve fibers, thereby increasing the release of the neurotransmitter noradrenaline.

Presynaptic receptors have been the focus of Klaus Starke’s scientific work since his habilitation. His research significantly contributed to clarifying the question of how antagonists at α_2_-adrenoceptors increase the release of noradrenaline from sympathetic nerves. Klaus Starke showed that these antagonists block presynaptic α_2_-adrenoceptors, which normally inhibit transmitter release. Presynaptic α_2_-adrenoceptors are part of a feedback loop that controls sympathetic noradrenaline release. This regulatory principle has been identified for many other transmitters and is one of the basic principles of neurotransmitter release in the central and peripheral nervous system.

In 1977, Klaus Starke was appointed to a professorship at the University of Freiburg to head the newly established Division of Molecular Pharmacology within the Institute of Pharmacology. Despite further offers for professorships in Bonn, Essen, and Würzburg, Germany, Klaus Starke remained Head of the Department of Pharmacology in Freiburg until his retirement in 2003.

His research findings on presynaptic receptors were mostly published in *Naunyn–Schmiedeberg’s Archives of Pharmacology*. To date, more than 300 papers on the subject have been published here, many of them by Klaus Starke. For many years, Klaus Starke was co-editor (1976–2003) and managing editor (1986–1994) of the journal and shaped its development. In 1988, on the occasion of the 125th anniversary of *Naunyn–Schmiedeberg’s Archives of Pharmacology*, Klaus Starke published an article on the history of the journal that is well worth reading (Starke 1998).

Klaus Starke shaped *Naunyn–Schmiedeberg’s Archives of Pharmacology* like no other scientist. In the more than 150-year history of the journal, he is by far the author with the most publications (> 120), and with his work he ensured that the Institute of Experimental and Clinical Pharmacology and Toxicology at the University of Freiburg became a “hotspot” of pharmacological research in Germany (Dats et al. 2023).

Several papers of Klaus Starke are among the most cited publications of the journal (Dats et al. 2023), which impressively underlines his high international reputation.

Klaus Starke was a devoted and highly talented academic teacher. His rousing lectures, peppered with exciting anecdotes about medicines and toxins in movies and cultural contexts captivated generations of medical and pharmacy students. His legendary end-of-year lectures shortly before Christmas filled the lecture hall, and students and colleagues alike listened spellbound to topics such as the cultural history of ergot from Saint Anthony’s fire to the molecular pharmacology of ergotamine. For more than 30 years, Klaus Starke was co-editor of the German textbook *Allgemeine und Spezielle Pharmakologie und Toxikologie* (Basic and Comprehensive Pharmacology and Toxicology).

In addition to pharmacological research and teaching, Prof. Starke was very active in academic self-administration at his faculty, university, and in national research organizations. From 1986 to 1987, he was Dean of the Medical Faculty in Freiburg. He was the founding spokesman of the Collaborative Research Center 325 “Modulation and Learning Processes in Neuronal Systems” of the German Research Foundation (DFG). From 1992 to 1998, he was a member of the Senate Committee for the DFG Collaborative Research Centers. It was thanks to Klaus Starke that both the Institute of Pharmacology and the Institute of Pharmacy of the University of Freiburg moved into a new building, the Otto Krayer House, in 2001.

Klaus Starke has received many honors for his scientific achievements. He was elected a member of the German National Academy of Sciences Leopoldina in 1987, a member of the Academia Europaea in 1991, and a member of the Heidelberg Academy of Sciences and Humanities in 1994. He was an honorary member of the British, German, and Hungarian Pharmacological Societies. In 2003, he received the Medal of Honor from the University of Freiburg.

In 2023, Klaus Starke was awarded the Schmiedeberg Medal of the German Society for Experimental and Clinical Pharmacology and Toxicology (DGPT) on the occasion of the annual meeting of the society in Ulm, Germany. The laudation stated “The DGPT honors Prof. Starke with the Schmiedeberg medal for his outstanding contributions to elucidate the function of presynaptic receptors, his co-editorship of the classic German textbook *Allgemeine und Spezielle Pharmakologie und Toxikologie* and his contributions to the scientific journal of the DGPT, the *Naunyn–Schmiedeberg’s Archives of Pharmacology*.”

We have lost an outstanding scientist, deeply dedicated to basic research and teaching, and a highly esteemed colleague.

Our heartfelt condolences go out to his family.
